# Murine CXCR3^+^CXCR6^+^γδT Cells Reside in the Liver and Provide Protection Against HBV Infection

**DOI:** 10.3389/fimmu.2021.757379

**Published:** 2022-01-21

**Authors:** Yanan Wang, Yun Guan, Yuan Hu, Yan Li, Nan Lu, Cai Zhang

**Affiliations:** ^1^ Institute of Immunopharmaceutical Sciences, School of Pharmaceutical Sciences, Cheeloo College of Medicine, Shandong University, Jinan, China; ^2^ Jining No. 1 People’s Hospital, Jining, China; ^3^ Institute of Biomedical Sciences, College of Life Sciences, Shandong Normal University, Jinan, China; ^4^ Institute of Diagnostics, School of Medicine, Cheeloo College of Medicine, Shandong University, Jinan, China

**Keywords:** liver, γδT cells, residency, liver-resident γδT cells, chemotaxis, HBV infection

## Abstract

Gamma delta (γδ) T cells play a key role in the innate immune response and serve as the first line of defense against infection and tumors. These cells are defined as tissue-resident lymphocytes in skin, lung, and intestinal mucosa. They are also relatively abundant in the liver; however, little is known about the residency of hepatic γδT cells. By comparing the phenotype of murine γδT cells in liver, spleen, thymus, and small intestine, a CXCR3^+^CXCR6^+^ γδT-cell subset with tissue-resident characteristics was found in liver tissue from embryos through adults. Liver sinusoidal endothelial cells mediated retention of CXCR3^+^CXCR6^+^ γδT cells through the interactions between CXCR3 and CXCR6 and their chemokines. During acute HBV infection, CXCR3^+^CXCR6^+^ γδT cells produced high levels of IFN-γ and adoptive transfer of CXCR3^+^CXCR6^+^ γδT cells into acute HBV-infected TCRδ^−/−^ mice leading to lower HBsAg and HBeAg expression. It is suggested that liver resident CXCR3^+^CXCR6^+^ γδT cells play a protective role during acute HBV infection. Strategies aimed at expanding and activating liver resident CXCR3^+^CXCR6^+^ γδT cells both *in vivo* or *in vitro* have great prospects for use in immunotherapy that specifically targets acute HBV infection.

## Introduction

The liver is not only involved in regulating metabolism but also has important immunological characteristics (1, 2). Liver tissue is enriched with innate immune cells that can effectively and quickly defend against invading microorganisms and tumor transformation (3–5). Lymphocytes are classically viewed as continuously circulating in peripheral organs; however, recent studies demonstrate the existence of tissue-resident lymphocytes in the skin, lung, and intestinal mucosa and other peripheral organs, where they exert protective immunity to infection and malignancy (6–9). Notably, the liver contains multiple resident lymphocytes including memory CD8^+^ T (T_RM_) cells, invariant natural killer T (iNKT) cells, mucosal-associated invariant T (MAIT) cells, and natural killer (NK) cells (10, 11). Liver-resident lymphocytes have a similar distribution, phenotype, and method of transcriptional regulation and function (12–15).

γδT cells are a unique subset of innate-like T lymphocytes with diverse structural and functional heterogeneity. Unlike αβT cells, γδT cells recognize antigens independent of MHC restriction and do not require antigen processing and presentation (16, 17). Murine γδT cells constitute only a small proportion of lymphocytes in peripheral organs—such as spleen and lymph nodes—but are abundant in the liver, accounting for 3%–5% of all intrahepatic lymphocytes. In an acute HBV infection model, the number of hepatic γδT cells significantly increases and exhibits elevated expression of the activation marker, CD69 (18). However, the role of γδT cells during the early stages of acute HBV infection is not well defined. γδT cells are present in high numbers in epithelial and mucosal barriers and several γδT-cell subsets naturally establish residency at barrier sites, as illustrated by the presence of Vγ5^+^ and Vγ3Vδ1^+^ T-cell subsets in the intestinal epithelium and epidermis, respectively (19–22). Tian’s group reported a liver-resident γδT population maintained by gut commensal microbes through CD1d/lipid antigens. This liver-resident γδT-cell population predominantly produced IL-17A and accelerated the progression of nonalcoholic fatty liver disease (23). However, there is a limited understanding of the resident characteristics of γδT cells in the hepatic immune microenvironment. A deeper and more comprehensive understanding of liver-resident γδT cells will inform the development of novel treatments for diverse liver diseases.

## Materials and Methods

### Animals

Five to 8-week-old male C57BL/6J, BALB/c, and nude mice were purchased from Beijing Hua Fukang Bioscience Co., Ltd. (Beijing, China). TCRδ^−/−^ mice were a gift from Dr. Zhinan Yin (Nankai University). CD45.1^+^ mice were a gift from Dr. Zhigang Tian (University of Science and Technology of China). All mice were housed in a specific pathogen-free facility under ethical conditions. Experiments were performed with age- and sex-matched animals according to the guidelines for experimental animals from Shandong University and were approved by the Committee on the Ethics of Animal Experiments of Shandong University (Jinan, China) (2017-D023).

### Acute HBV Infection Model

The pAAV plasmid and the pAAV-HBV1.2 plasmid containing the full-length HBV DNA were kindly provided by Dr. Peijer Chen (National Taiwan University College of Medicine, Taipei, China). An acute HBV infection model was established by hydrodynamic injection of 20 μg pAAV-HBV1.2 plasmid in 1× phosphate buffer solution (PBS) equivalent to 10% of the mouse body weight by way of the tail veil into wild-type (WT) C57BL/6J and TCRδ^−/−^ mice (24).

### Parabiosis

Parabiosis was established as described previously (25). In brief, 5- to 6-week-old male CD45.1^+^ and CD45.2^+^ C57BL/6J mice were matched for body weight and anaesthetized prior to surgery. An incision along the lateral aspect was performed from 0.5 cm above the elbow to 0.5 cm below the knee joint, and the skin was gently detached from the subcutaneous fascia. The knee joints and skins of the paired mice were attached from the elbow to the knee with nonabsorbable silk. Neomycin was added to the drinking water for 7 days. Two weeks after parabiosis, two mice shared the blood circulation.

### Cell Isolation

Mice were sacrificed, and the bone marrow (BM), inguinal lymph node (iLN), spleen, liver, thymus, intestine, and skin were collected. MNCs from each organ were separated and mononuclear cells (MNCs) were collected. In brief, iLN and thymus were passed through a 200-gauge steel mesh and centrifuged at 400×*g* for 5 min in 1 × PBS. Spleens were first passed through a 200-gauge steel mesh, lysed with red blood cell (RBC) lysis buffer and washed with 1 × PBS. Bone marrows were washed by syringe, and MNCs were harvested after RBC lysis and washing. Livers were passed through a 200-gauge steel mesh, and the cell pellets were collected. MNCs were isolated by gradient centrifugation with 40% Percoll at 800×*g* for 25 min and harvested after RBC lysis and washing. Skins were excised and minced into small pieces, then digested with 0.04% collagenase I (Gibco, Carlsbad, CA, USA) for 2 h at 37°C. The large pieces were removed by filtration, and the MNCs were obtained by gradient centrifugation with 40% and 70% Percoll. IELs were isolated from the small intestine as previously described (26, 27). Peyer’s patches (PP) were surgically removed from the intestines and excised into small pieces. The specimens were then digested with prewarmed IEL digestive juice and incubated with stirring for 40 min at 37°C and passed over two nylon wool columns to remove undigested tissue debris. IELs were isolated by gradient centrifugation with 40% and 70% Percoll (GE Healthcare, Uppsala, Sweden).

The mice were anesthetized prior to a laparotomy to obtain the LSECs. The liver was perfused with EGTA/HBSS solution through the portal vein. The inferior vena cava was rapidly cut after the liver turned completely pale. Next, the liver was perfused with 37°C prewarmed Collagenase IV (Gibco) solution for 5 min, excised, and digested in collagenase solution for 15 min in a 37°C incubator. LSECs were obtained by gradient centrifugation with 25% and 50% Percoll. The cellular precipitate was resuspended in 1 ml DMEM+10% FBS medium and seeded into 24-well plates at a density of 1 × 10^5^ cells/well. After 4 h, the supernatant was gently removed and replaced with 500 μl fresh DMEM medium.

### Flow Cytometry

MNCs in a single-cell suspension were blocked with anti-CD16/32 (eBioscience, San Diego, CA, USA) and stained with a cocktail of antibodies specific for different cell types for 30 min at 4°C. The foxp3 staining kit (eBioscience) was used to stain the nucleus transcription factor. To detect expression of IL-17A and IFN-γ, the cells were stimulated with phorbol 12-myristate 13-acetate (PMA, 30 ng/ml, Sigma-Aldrich Co., St. Louis, MO, USA) and ionomycin (1 μg/ml, Sigma-Aldrich) for 4 h and treated with monensin (1 μg/ml, Sigma-Aldrich) and brefeldin A (BFA, 1 μg/ml, Biolegend, San Diego, CA, USA) after 1 h of stimulation. After surface staining, cells were fixed, permeabilized, and stained with the indicated intracellular antibodies. Flow cytometry was performed using a fluorescence-activated cell sorter (FACS) Aria III (BD Biosciences, San Jose, CA, USA), and data were analyzed with FlowJo software (TreeStar Inc., Ashland, OR, USA). At least 3 × 10^5^ total events were needed for the FACS analysis. Data are presented in **Supplementary Table S1**.

### Cell Sorting and Adoptive Transfer

Hepatic MNCs were stained with anti-CD3e, anti-TCRγ/δ, anti-CXCR3, and anti-CXCR6 antibodies to sort CXCR3^+^CXCR6^+^ γδT cells using a FACS Aria III (BD Biosciences). The purity of the sorted cell populations was >95%. Approximately 50,000 purified CXCR3^+^CXCR6^+^ γδT cells in 200 μl 1 × PBS were intravenously injected into sublethally irradiated TCRδ^−/−^ mice (6.5 Gy given 1 day before adoptive transfer). An acute HBV infection model was established in WT, TCRδ^−/−^, and TCRδ^−/−^ mice after adoptive transfer of CXCR3^+^CXCR6^+^ γδT cells.

### RNA Isolation and Real-Time PCR

Total γδT-cell RNA from the indicated tissues was extracted using TRIZOL reagent (Invitrogen, Carlsbad, CA, USA) according to the manufacturer’s instructions. The RNA concentration was quantified using Nanodrop 2000 (Thermo Fisher, Waltham, MA, USA). cDNA was generated using a FastQuant RT Kit (Tiangen Biotech Co. Ltd., Beijing, China) and real-time polymerase chain reaction was performed using a SYBR Green SuperMix (Roche, Basel, Switzerland) on a LightCycler 480 platform (Roche). Relative mRNA levels were normalized to β-actin mRNA levels. The primers are described in **Supplementary Table S2**.

### Transwell Assay

Hepatic MNCs were seeded at 5 × 10^5^ in 100 μl serum-free RPMI-1640 media in the above inserts with a pore size of 0.4 μm. The lower chamber contained 500 μl DMEM medium with untreated LSECs or LSECs incubated with neutralizing chemokine-targeting antibodies at 37°C for 2 h. After a 2-h incubation, absolute numbers of the migrated cells were detected by FACSAria III.

### Chemokine Detection by ELISA

The LSECs were inoculated into 24-well plates (1 × 10^5^ cells/well). After culturing for 4 h, the supernatant was absorbed, washed three times with 1 × PBS, and replaced with fresh DMEM medium (500 μl). Cell culture supernatants were collected at 24 h. CXCL9, CXCL10, CXCL11, and CXCL16 chemokine levels were detected in the LSEC culture supernatants by ELISA (PeproTech, East Windsor, New Jersey, USA) according to the manufacturers’ instructions.

### Serum HBV Ag Assays

Serum hepatitis B surface Ag (HBsAg) and hepatitis BeAg (HBeAg) from mice with acute HBV infection were assayed using the HBsAg and HBeAg detection kits (Autobio, Zhengzhou, China) according to the manufacturer’s instructions.

### HBV DNA Detection

Serum HBV DNA copies were extracted from 50 μl serum and detected by quantitative PCR using a diagnostic kit for HBV DNA (Da An Gene, Guangzhou, China) according to the manufacturer’s instructions.

### Statistical Analysis

The data were analyzed using GraphPad Prism 7 software (GraphPad Software Inc., San Diego, CA, USA). Data are presented as the mean ± standard error of the mean (SEM). Differences between more than two groups were statistically analyzed by one-way analysis of variance (one-way ANOVA). A two-way ANOVA test was used to determine differences in HBsAg, HBeAg, and HBV DNA tests. A *p*-value <0.05 was considered statistically significant (^*^ *p* < 0.05; ^**^ *p* < 0.01; ^***^ *p* < 0.001).

## Results

### CXCR3^+^CXCR6^+^ γδT Cells Only Exist in Liver

**Figure 1:**
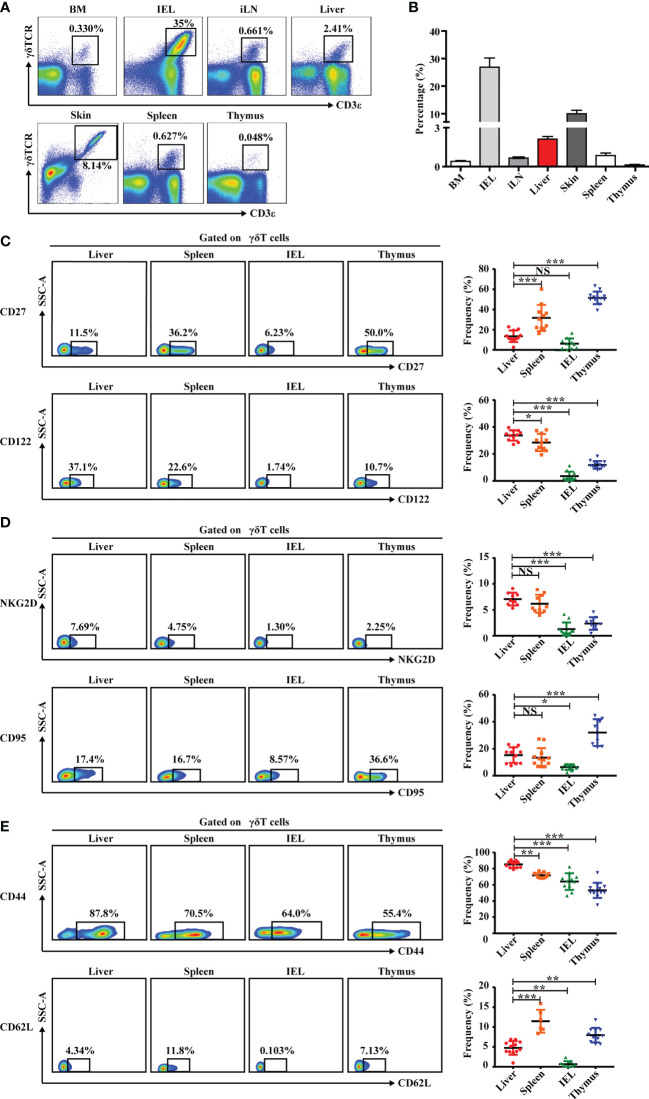
Liver contains abundant γδT cells that exhibit a unique phenotype. **(A)** Representative FACS plots of γδT cells (CD3^+^TCRγδ^+^) in BM, IEL, iLN, liver, skin, spleen, and thymus of C57BL/6J mice. **(B)** Statistical analysis of γδT-cell percentages in the indicated organs. **(C–E)** Representative FACS plots (left) and percentage (right) of γδT surface markers on cells in indicated organs. Each dot represents a mouse. Data are shown as mean ± SEM. (^*^ *p* < 0.05; ^**^ *p* < 0.01; ^***^ *p* < 0.001. One-way ANOVA with *post-hoc* test). BM, bone marrow; IEL, intraepithelial lymphocyte; iLN, inguinal lymph node; NS, not significant.

IELs and skin are rich in γδT cells, where they regulate inflammation, pathogen invasion, and tumor surveillance (22, 28, 29). In this study, the liver also had a higher proportion of γδT cells than other immune organs and tissues, including the BM, inguinal LN, spleen, and thymus (**Supplementary Figure S1** and **Figures 1A, B**). This finding correlates with results from a recent study (23).

This study also assessed the phenotype of γδT cells in liver, first by analyzing molecules associated with γδT-cell-induced cytokine production. Hepatic γδT cells expressed low levels of CD27, which is a fate determinant of γδT cells to express IFN-γ but not IL-17A. The expression was similar in γδT cells from the IEL but significantly lower than γδT cells from the spleen and thymus (30) (**Figure 1C**). Hepatic γδT cells also expressed higher levels of IL-2 receptor beta CD122, which is expressed on IFN-γ^+^ γδT cells, compared with γδT cells from other immune organs and tissues (31) (**Figure 1C**). Hepatic γδT cells exhibited higher NKG2D expression, indicating that these cells may be involved in immune defense and tissue protection in the liver (**Figure 1D**). γδT cells induce effector−target cell apoptosis through the Fas–FasL pathway (32, 33). Hepatic γδT cells have a higher expression of Fas (CD95) than intestinal epithelial γδT cells but lower expression than thymic γδT cells (**Figure 1D**). Importantly, hepatic γδT cells expressed a high level of the adhesion and retention molecule, CD44, and a low level of the lymph node homing molecule, CD62L (**Figure 1E**). Overall, these data indicate that hepatic γδT cells exhibited a unique phenotype that was distinct from γδT cells found in other organs.

**Figure 2:**
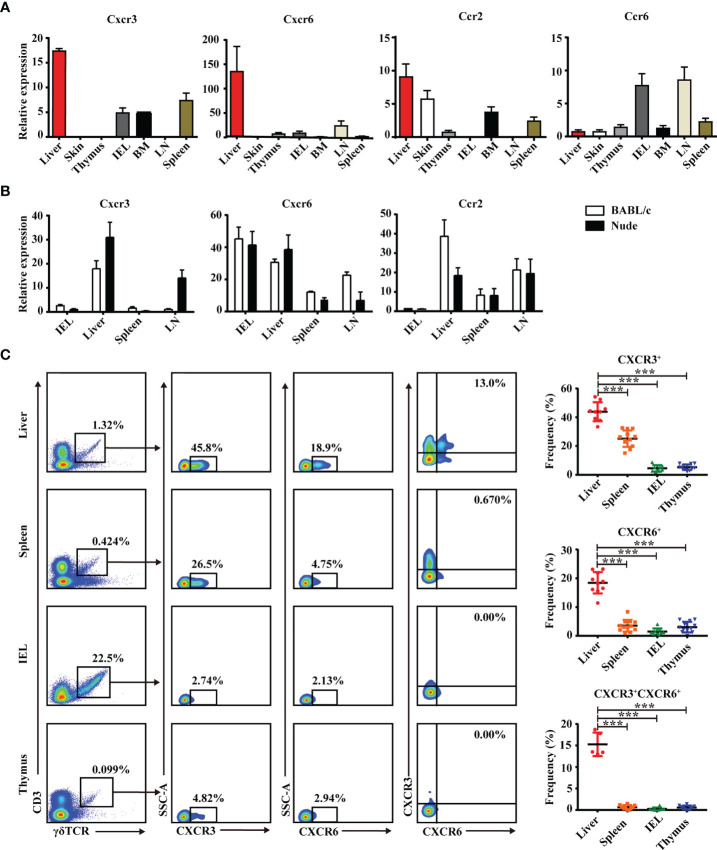
Liver contains a specific CXCR3^+^CXCR6^+^ γδT subset. **(A)** CXCR3, CXCR6, CCR2, and CCR6 mRNA expression in γδT cells from indicated organs of C57BL/6J mice. **(B)** CXCR3, CXCR6, and CCR2 mRNA expression in γδT cells from IEL, liver, spleen, and iLN of BALB/c and nude mice. **(C)** Representative FACS plots and percentage of γδT cells expressing CXCR6 or CXCR3 in liver, spleen, IELs, and thymus of C57BL/6J mice. Each dot represents a mouse. Data are shown as mean ± SEM. (^***^ *p* < 0.001. One-way ANOVA with *post-hoc* test).

Chemokines and chemokine receptors regulate the trafficking and accumulation of lymphocytes in homeostasis and disease. The mRNA level of chemokine receptors in γδT cells was measured in multiple organs. As shown in **Figure 2A**, CXCR3, CXCR6, and CCR2 mRNA levels were higher in hepatic γδT cells than in other tissues and organs. Hepatic γδT cells from nude mice still retained high CXCR3 and CXCR6 expression (**Figure 2B**), indicating that thymus deficiency did not impact expression. CXCR3 and CXCR6 expression was further examined by flow cytometry. Results showed that hepatic γδT cells expressed higher levels of CXCR3 and CXCR6 than those from spleen, IEL, and thymus in C57BL/6J mice (**Supplementary Figure S2** and **Figure 2C**). Furthermore, the CXCR3^+^CXCR6^+^ γδT-cell subset was found in liver but not in the spleen, IEL, or thymus (**Figure 2C**). These data indicate that hepatic γδT cells exhibited a unique phenotype and the CXCR3^+^CXCR6^+^ γδT subset specifically exists in liver.

### CXCR3^+^CXCR6^+^ γδT Cells Have Tissue-Resident Characteristics and Specifically Reside in Liver

**Figure 3:**
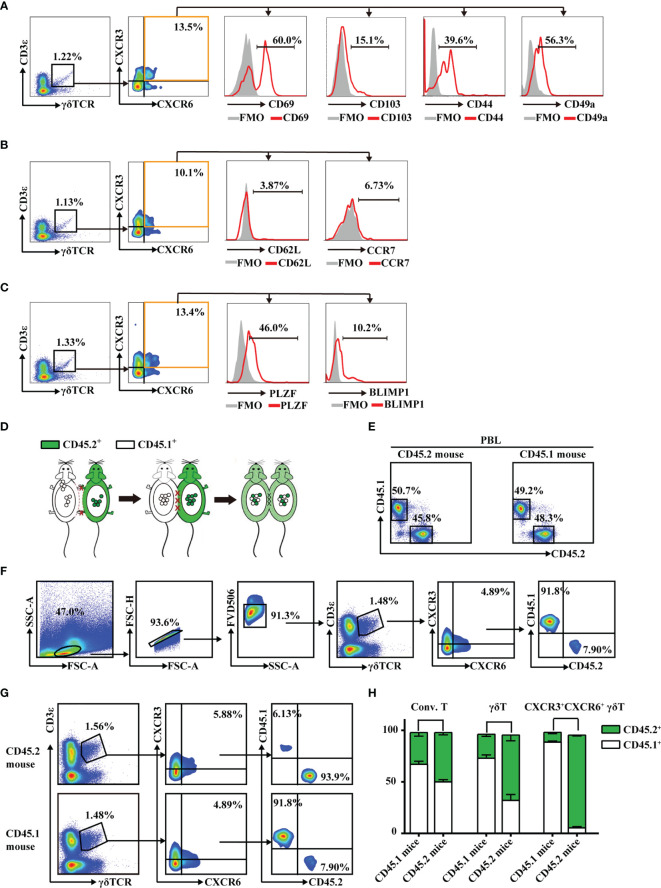
CXCR3^+^CXCR6^+^ γδT cells have tissue-resident characteristics and specifically reside in liver. **(A–C)** FACS analysis of CD69, CD103, CD44, and CD49a **(A)**, CD62L and CCR7 **(B)**, and PLZF and Blimp1 **(C)** expression on hepatic CXCR3^+^CXCR6^+^ γδT cells from C57BL/6J mouse livers. **(D)** Parabiotic joining of CD45.1 and congenic CD45.2 mice led to the development of a shared circulatory system 2 weeks after surgery. **(E)** The percentages of CD45.1^+^ and CD45.2^+^ cells in peripheral blood lymphocytes (PBLs) in parabiosis models. **(F)** Gating strategy of CD45.1^+^ or CD45.2^+^ expression on hepatic CXCR3^+^CXCR6^+^ γδT cells. **(G)** The host origin (CD45.1^+^ or CD45.2^+^) of hepatic CXCR3^+^CXCR6^+^ γδT cells was identified by FACS analysis in each mouse in the CD45.1/CD45.2 parabiotic mouse pairs at 14 days postsurgery. **(H)** The percentage of CD45.1^+^ or CD45.2^+^ conventional T cells (CD3^+^TCRγδ^-^NK1.1^−^), γδT cells (CD3^+^TCRγδ^+^), and CXCR3^+^CXCR6^+^ γδT cells from the liver of CD45.1/CD45.2 parabiotic B6 mouse.

Tissue-resident lymphocytes often express CD103, CD69, CD44, and CD49a, which mediate adhesion and retention, and lack the lymphoid homing markers, CD62L and CCR7 (6, 34). In this study, hepatic CXCR3^+^CXCR6^+^ γδT cells expressed high levels of CD69, CD103, CD44, and CD49a (**Figure 3A**) and low levels of CD62L and CCR7 (**Figure 3B** and **Supplementary Figure S3**). These cells also expressed a high level of the transcription factor, PLZF, which is important for innate tissue-resident lymphocytes like iNKT cells and MAIT cells (35), and a low level of BlIMP1, which correlates with diverse tissue-resident lymphocyte populations such as T_RM_ and NKT cells (**Figure 3C**). These data indicate that hepatic CXCR3^+^CXCR6^+^ γδT cells have tissue-resident characteristics.

To further confirm whether these cells specifically reside in liver, CD45.1^+^ and CD45.2^+^ mice were surgically joined by parabiosis (**Figure 3D**). After 2 weeks, the percentages of CD45.1^+^ or CD45.2^+^ cells in peripheral blood lymphocytes (PBLs) were equivalent, indicating that the parabiosis models were successfully established (**Figure 3E**). The redistribution of γδT cells in each mouse in the CD45.1/CD45.2 parabiotic mouse pairs was assessed (**Figure 3F**). While conventional T cells were mutually exchanged, hepatic CXCR3^+^CXCR6^+^ γδT cells from the CD45.1^+^ parabiont mice were almost entirely CD45.1^+^, with very few CD45.2^+^, while CXCR3^+^CXCR6^+^ γδT cells from the CD45.2^+^ parabiont mice were primarily CD45.2^+^ (**Figures 3G, H**). These data suggest that hepatic CXCR3^+^CXCR6^+^ γδT cells were seldom exchanged between CD45.1^+^ and CD45.2^+^ parabiont mice. In addition, 70%–80% CXCR3^+^CXCR6^−^ γδT cells and CXCR3^−^CXCR6^+^ γδT cells were CD45.1^+^ in CD45.1^+^ parabiont mice, whereas the reverse was observed in CD45.2^+^ parabiont mice (**Supplementary Figure S4**). In summary, the parabiosis assay indicated that CXCR3^+^CXCR6^+^ γδT cells were predominantly retained in the liver, while CXCR3^+^CXCR6^−^ γδT cells and CXCR3^−^CXCR6^+^ γδT cells have less liver-resident features.

### Liver-Resident CXCR3^+^CXCR6^+^ γδT Cells Exist in Liver From Embryo to Adulthood

**Figure 4:**
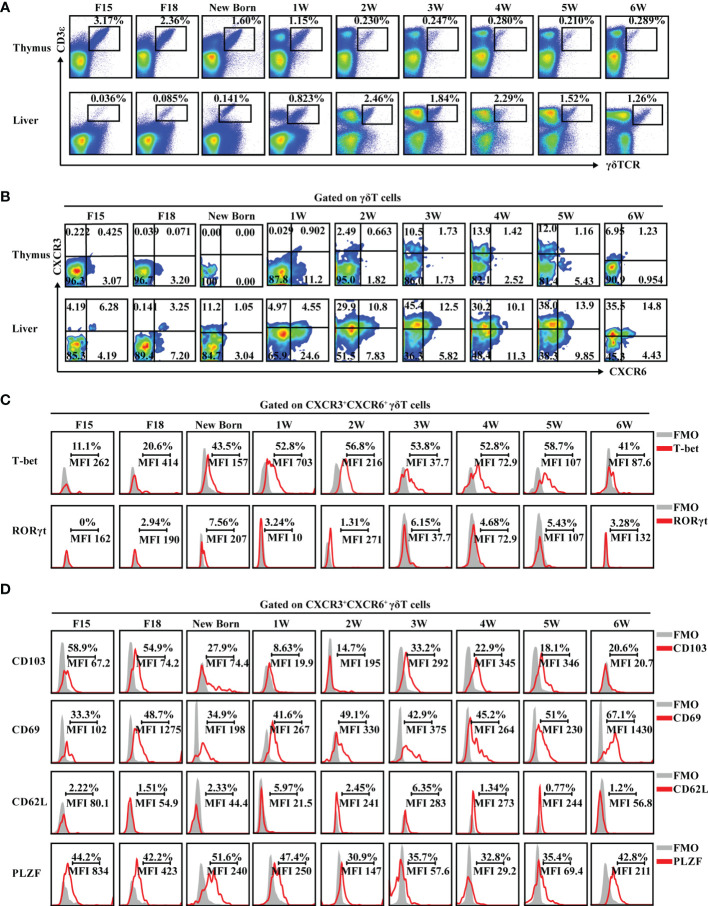
CXCR3^+^CXCR6^+^ γδT cells exist in liver and express tissue-resident markers from embryo to adulthood. **(A)** FACS analysis of γδT cells in thymus (top) and liver (bottom) at different developmental stages of C57BL/6J mice. **(B)** FACS analysis of CXCR3^+^CXCR6^+^ γδT cells in the thymus and liver at the different developmental stages of C57BL/6J mice. **(C, D)** FACS analysis of T-bet and RORγt **(C)** and CD103, CD69, CD62L, and PLZF expression **(D)** in hepatic CXCR3^+^CXCR6^+^ γδT cells at the indicated ages over time.

In general, γδT cells are exported from the thymus at defined periods of fetal and neonatal development, and then migrate to and populate different peripheral tissues in adult animals (36). However, intestinal γδT cells can also develop in intestinal crypts and reside in the gut (37). To explore whether the existence of hepatic γδT cells was dependent on the thymus, the proportion of γδT cells in athymic nude and WT BALB/c mice were assessed. The proportion of intestinal γδT cells in nude mice was nearly identical to the proportion of intestinal γδT cells in WT BALB/c mice. Importantly, the liver still contained γδT cells in athymic nude mice despite the lack of γδT cells in the spleen (**Supplementary Figures S5A, B**). These data suggest that some hepatic γδT cells are not dependent on the thymus. Assessment of γδT cells during different stages of growth showed that they are found in liver and thymus from embryo to adulthood. As the mice grew, the percentage of γδT cells decreased in the thymus and increased in the liver (**Figure 4A** and **Supplementary Figure S6**). More importantly, CXCR3^+^CXCR6^+^ γδT cells were present in the liver from embryo to adulthood and the proportion increased as the mice aged; however, they were absent in the thymus during particular stages of growth (**Figure 4B**).

CXCR3^+^CXCR6^+^ γδT cells expressed high levels of the IFN-γ-associated transcriptional factor, T-bet, from embryo to adulthood, while rarely expressing the IL-17A-associated transcriptional factor, RORγt (**Figure 4C**). In addition, hepatic CXCR3^+^CXCR6^+^ γδT cells expressed a high level of tissue-resident-associated molecules like CD103 and CD69 and the transcription factor, PLZF, but rarely expressed CD62L during development (**Figure 4D** and **Supplementary Figure S7**).

In summary, these data collectively indicate that CXCR3^+^CXCR6^+^ γδT cells are retained in liver from embryo to adulthood and have tissue-resident characteristics during mouse development.

### Liver Sinusoidal Endothelium Cells Mediate Retention of Liver-Resident CXCR3^+^CXCR6^+^ γδT Cells

**Figure 5:**
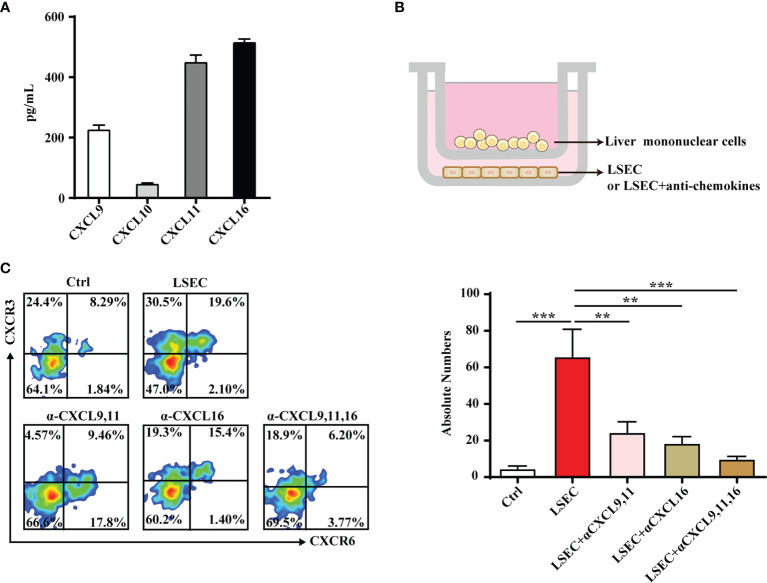
LSECs promote the recruitment of CXCR3^+^CXCR6^+^ γδT cells to the liver. **(A)** Freshly isolated LSECs (1 × 10^5^ per well) were seeded into 24-well plates and incubated at 37°C. The culture supernatant of each well was collected at 24 h and CXCL9, CXCL10, CXCL11, and CXCL16 levels in the culture supernatant were detected by ELISA. **(B)** Transwell was performed to test the chemotactic effect of LSECs to CXCR3^+^CXCR6^+^ γδT cells. The inserts were seeded with 5 × 10^5^ liver mononuclear cells in 100 µl serum-free RPMI-1640 media. The lower chamber contained 500 µl DMEM medium with untreated LSECs or LSECs incubated with neutralizing antibodies specific for indicated chemokines at 37°C for 2 h. **(C)** FACS analysis (left) and absolute numbers (right) of CXCR3^+^CXCR6^+^ γδT cells in the lower chamber migrated from the upper insert after a 2-h incubation. Data are shown as mean ± SEM. (^**^ *p* < 0.01; ^***^ *p* < 0.001. One-way ANOVA with *post-hoc* test).

LSECs secrete a variety of chemokines that promote the recruitment of immune cells (38, 39). Results from the present study showed that the liver-resident γδT-cell subset specifically expressed the chemokine receptors, CXCR3 and CXCR6. Thus, we hypothesized that chemotaxis may play an important role in the recruitment and retention of CXCR3^+^CXCR6^+^ γδT cells in the liver. LSECs were isolated and chemokines, including the ligands of CXCR3 (CXCL9, CXCL10, CXCL11) and the ligand of CXCR6 (CXCL16), were measured in the culture supernatants. LSECs produced high levels of CXCL9, CXCL11, and CXCL16 (**Figure 5A**). To further demonstrate the recruitment function of LSECs, a transwell assay was performed to test the chemotactic ability of chemokines secreted by LSECs on CXCR3^+^CXCR6^+^ γδT cells (**Figure 5B**). When the lower chamber contained untreated LSECs, CXCR3^+^CXCR6^+^ γδT cells were recruited from the above inserts. Chemotaxis was significantly reduced when CXCL9 and CXCL11 were neutralized, indicating that the chemokines, CXCL9 and CXCL11, play important roles in the retention of CXCR3^+^CXCR6^+^ γδT cells (**Figure 5C**). When CXCL16 was neutralized, the level of chemotaxis between the LSECs and CXCR3^+^CXCR6^+^ γδT cells was also weakened (**Figure 5C**). These findings indicate that the chemokines, CXCL9, CXCL11, and CXCL16, are all required for the retention of CXCR3^+^CXCR6^+^ γδT cells. Taken together, these results indicate that LSECs play an important role in retaining liver-resident CXCR3^+^CXCR6^+^ γδT cells through the interaction between the chemokine receptors, CXCR3 and CXCR6, and their ligands.

### Liver-Resident CXCR3^+^CXCR6^+^ γδT Cells Play a Protective Role During Acute HBV Infection

**Figure 6:**
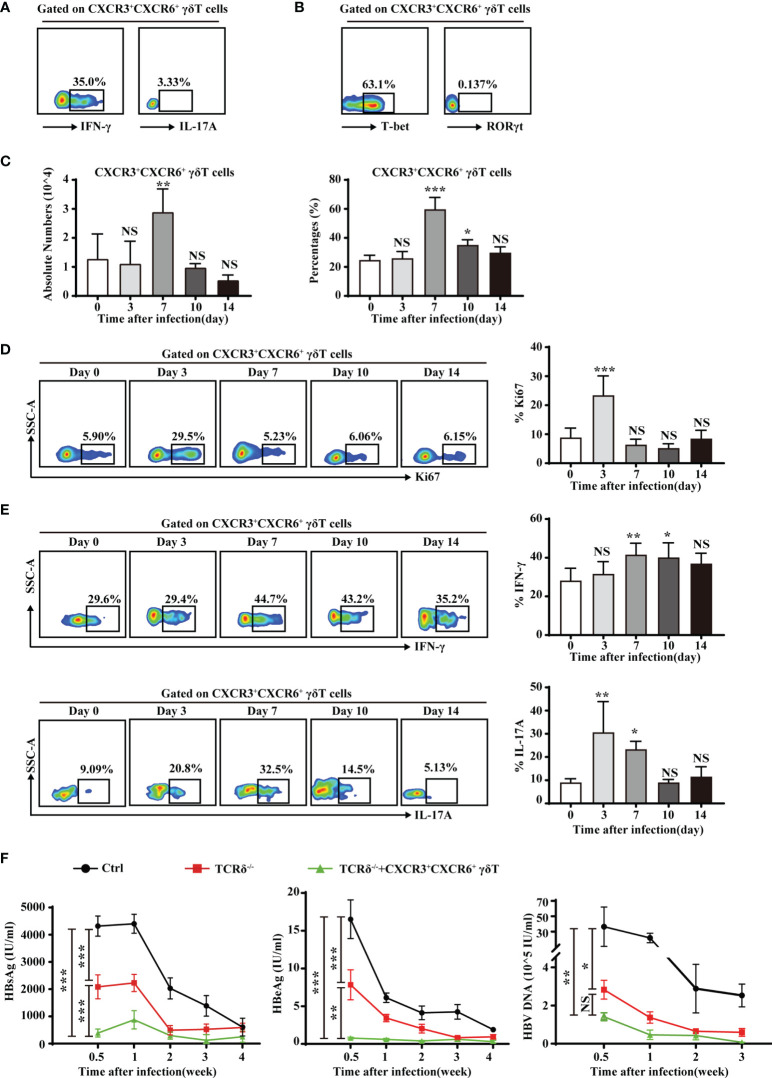
Liver-resident CXCR3^+^CXCR6^+^ γδT cells contribute to the clearance of HBV. **(A)** IFN-γ and IL-17A expression on PMA/ionomycin-stimulated CXCR3^+^CXCR6^+^ γδT cells in the liver of C57BL/6J mice. **(B)** T-bet and RORγt expression in hepatic CXCR3^+^CXCR6^+^ γδT cells. **(C)** An acute HBV infection model was established by hydrodynamic injection of pAAV-HBV1.2 plasmid into the tail veil. The absolute numbers (left) and percentages (right) of liver-resident CXCR3^+^CXCR6^+^ γδT cells after acute HBV infection at the indicated time points. **(D)** FACS analysis (left) and statistical analysis (right) of Ki67 on CXCR3^+^CXCR6^+^ γδT cells after acute HBV infection at the indicated time points. **(E)** FACS analysis (left) and statistical analysis (right) of IFN-γ (top) and IL-17 (bottom) secreted by PMA/ionomycin-stimulated CXCR3^+^CXCR6^+^ γδT cells after acute HBV infection at the indicated time points. **(E)** HBsAg, HBeAg, and HBV DNA detection in serum at the indicated time points of an HBV acute infection model in WT, TCRδ^−/−^, and TCRδ^−/−^ mice after adoptive transfer of CXCR3^+^CXCR6^+^ γδT cells. Data are shown as mean ± SEM. [^*^ *p* < 0.05; ^**^ *p* < 0.01; ^***^ *p* < 0.001. One-way ANOVA with *post-hoc* test **(C–E)**, two-way ANOVA test **(F)**]. NS, not significant.

Liver-resident CXCR3^+^CXCR6^+^ γδT cells produced high levels of IFN-γ and expressed a high level of the IFN-γ transcription factor, T-bet (**Supplementary Figure S8** and **Figures 6A, B**). However, these cells produced very little IL-17A and expressed low levels of the IL-17A transcription factor, RORγt (**Figures 6A, B**). The production of IFN-γ indicated that liver-resident CXCR3^+^CXCR6^+^ γδT cells may be involved in protective immunity against viruses and intracellular pathogens. In an acute HBV infection model, the proportion and absolute numbers of CXCR3^+^CXCR6^+^ γδT cells increased on day 7 and returned to normal levels as the infection progresses (**Figure 6C**). The expression of Ki67 was measured at different time points. On the third day after infection, CXCR3^+^CXCR6^+^ γδT cells showed significantly higher expression of Ki67, indicating that the proliferative capacity of CXCR3^+^CXCR6^+^ γδT cells was enhanced postinfection (**Figure 6D**). Notably, IFN-γ levels increased after 7 days postinfection (**Figure 6E**). These results indicate that liver-resident CXCR3^+^CXCR6^+^ γδT cells proliferate during acute HBV infection and IFN-γ from these cells may be involved in eliminating HBV. IL-17A secretion increased at the early stage of infection, and decreased after 10 days, and was then maintained at a low level (**Figure 6E**).

To further investigate the role of CXCR3^+^CXCR6^+^ γδT cells during HBV infection, the acute HBV infection model was established in WT, TCRδ^−/−^ mice, and TCRδ^-/-^ mice that were adoptively transferred with CXCR3^+^CXCR6^+^ γδT cells. HBsAg, HBeAg, and HBV DNA levels were measured in serum at different time points. TCRδ^−/−^ mice that had received CXCR3^+^CXCR6^+^ γδT cells exhibited markedly lower serum HBsAg, HBeAg, and HBV DNA levels than TCRδ^−/−^ mice that did not receive CXCR3^+^CXCR6^+^ γδT cells (**Figure 6F**). These data suggest that liver-resident CXCR3^+^CXCR6^+^ γδT cells may be involved in the clearance of HBV. Interestingly, TCRδ^−/−^ mice had lower serum HBsAg, HBeAg, and HBV DNA than WT mice, suggesting that other subsets of γδT cells may have different or even opposing effects during acute HBV infection. The roles of other subsets of γδT cells in acute HBV infection deserved further research.

These findings demonstrate that liver-resident CXCR3^+^CXCR6^+^ γδT cells exert a protective role during acute HBV infection through production of IFN-γ.

## Discussion

In the present study, a unique γδT-cell subset characterized by CXCR3^+^CXCR6^+^ expression was specifically found to reside in murine liver. Moreover, these cells existed from embryo to adult and exhibited tissue-resident characteristics. LSECs promoted the recruitment and retention of CXCR3^+^CXCR6^+^ γδT cells by secreting CXCL9, CXCL11, and CXCL16. Importantly, liver-resident CXCR3^+^CXCR6^+^ γδT cells secreted high levels of IFN-γ and provided protection against acute HBV infection.

Tissue-resident lymphocytes share similar phenotypes, functional properties, and transcriptional regulation (10, 34). CXCR3 and CXCR6 are present in liver-resident NK cells and CD8^+^ T cells (40–42). The engagement of chemokines and chemokine receptors is required for lymphocyte trafficking and retention in the liver. CXCR3 and its ligands mediate T-cell and TRAIL^+^ liver-resident NK cell chemotaxis toward the liver (43, 44). Liver-resident NK cells, NKT cells, and CD8^+^ T cells are selectively retained in liver in response to the chemotactic stimuli provided through the CXCR6 and CXCL16 interaction (45–47). Results from the present study provide the first evidence that CXCR3, CXCR6, and their ligands are critical for the accumulation and residency of γδT cells. Hepatic CXCR3^+^CXCR6^+^ γδT cells express PLZF, which is involved in a transcriptional network that promotes liver residency of human NK cells (48). A parabiosis model, one of the most common methods for assessing tissue residency (25), was used to further verify that CXCR3^+^CXCR6^+^ γδT cells specifically reside in the liver.

Similar to the intestine, the liver still contained γδT cells in athymic nude mice (**Supplementary Figure S5**), indicating that hepatic γδT cells do not dependent on the thymus. Furthermore, CXCR3^+^CXCR6^+^ γδT cells were not present in the thymus at all stages of growth, these cells were present in the liver from embryo to adulthood and the percentage increased as mice aged (**Figure 4**). These findings indicated that liver-resident CXCR3^+^CXCR6^+^ γδT cells may have a unique development pathway. While the thymus provides an inductive environment for the development of T cells from hematopoietic progenitor cells, prior studies have shown that γδT cells can develop extrathymically. Intestinal γδT cells, for example, can develop in intestinal crypts and reside in the gut (37, 49, 50). In addition, the adult liver contains hematopoietic stem and progenitor cells (HSPCs) and is believed to be an extramedullary hematopoietic organ (51–53). Hepatic heterogeneous Lin^−^Sca-1^+^c-Kit^+^ (LSK, contains hematopoietic stem cells and multipotent progenitors) cells can differentiate into both myeloid cells and lymphocytes, including γδT cells, and type 1 innate lymphoid cells (ILC1s) (54–56). Our recent findings showed that liver hematopoietic progenitor Lin^−^Sca-1^+^Mac-1^+^ (LSM) cells can differentiate into γδT-cell precursor (pre-γδT) cells and functionally mature IFN-γ^+^ γδT cells but not IL-17a^+^ γδT cells in a thymus-independent manner. This research suggests that γδT cells are involved in a distinct developmental pathway that is independent of the thymus (57). More evidence is required to better understand how liver-resident CXCR3^+^CXCR6^+^ γδT cells develop.

γδT cells can establish long-lived memory and resident populations in response to local inflammation or pathogen invasion and exert long-term protective roles (19–21, 58). Circulating γδT cells participate in lymphoid stress surveillance during HBV infection with differential activation and differentiation (59). However, the role of liver-resident γδT cells during acute HBV infection remains unknown. In the current study, CXCR3^+^CXCR6^+^ γδT cells produced more IFN-γ and were associated with reduced disease severity after adoptive transfer into HBV-infected TCRδ^−/−^ mice (**Figure 6**). These findings suggest that liver-resident CXCR3^+^CXCR6^+^ γδT cells inhibit acute HBV infection; however, the precise mechanism and memory characteristics of these cells require further study. In recent years, γδT cells have attracted more attention for potential use during tumor immunotherapy because of their strong cytotoxic effect on tumor cells, lack of MHC restriction on antigen recognition, and easy expansion both *in vitro* and *in vivo* (60–62). γδT cells are used for immunotherapy through *in vivo* stimulation with synthetic phosphoantigens like BrHPP, aminodicarbonates such as zoledronate, and glycolipid antigens presented by CD1d or adoptive transfer after expansion *ex vivo* (63–65). Current research is focused on combining direct antiviral agents and immunotherapy to treat HBV infection. Targeting the host immune system to strengthen innate and specific adaptive immune responses may help to eliminate this virus. DC vaccines, cytokine-induced killer (CIK) cells, immune checkpoint inhibitors, and genetically edited T cells (CAR/TCR-T) are used in the treatment of chronic viral infections and related cancers (66–68). In view of the antiviral effect of liver-resident CXCR3^+^CXCR6^+^ γδT cells, stimulation of these cells *in vivo* or adoptive transfer of liver-resident CXCR3^+^CXCR6^+^ γδT cells after *ex vivo* expansion may be a new therapeutic strategy for acute HBV infection.

Similar to mice, human livers also contain a liver-resident CD27^lo^CD45RA^lo^ subset of Vδ1^+^ γδT cells expressing high levels of CXCR3 and CXCR6. They produce significantly more of the pro-inflammatory cytokines, IFN-γ and TNF-α, and may be involved in responding to CMV infection (69). It is also worth noting that serum HBsAg, HBeAg, and HBV DNA levels are significantly lower in TCRδ^−/−^ mice than WT mice (**Figure 6F**). This may be related to the heterogeneity of γδT cells. γδT cells are a heterogeneous population and consist of different subsets including IFN-γ-producing γδ T cells, IL-17-producing γδ T cells, γδ NKT cells, and so on (57, 70). The TCRδ^−/−^ mice were systemic TCRδ gene deletion, and all γδT-cell subsets are deficient. Other γδT-cell subsets in liver may have different, even opposite, effects during acute HBV infection. The roles of other subsets of γδT cells in acute HBV infection deserved further research.

In summary, the current study was the first to demonstrate that CXCR3^+^CXCR6^+^ γδT cells are a liver-resident γδT-cell subset with a unique phenotype and tissue-resident characteristics. LSECs mediated the retention of CXCR3^+^CXCR6^+^ γδT cells through the interaction between the chemokine receptor, CXCR3, and its ligands, CXCL9 and CXCL11, as well as CXCR6 and its ligand, CXCL16. Liver-resident CXCR3^+^CXCR6^+^ γδT cells were protective against acute HBV infection. While additional research is required to better define the exact mechanism by which CXCR3^+^CXCR6^+^ γδT cells exert their protective role, findings from the current study provide more insight into liver-resident lymphocytes and provide novel strategies for the treatment of diverse liver diseases.

## Data Availability Statement

The raw data supporting the conclusions of this article will be made available by the authors, without undue reservation.

## Ethics Statement

The animal study was reviewed and approved by the Committee on the Ethics of Animal Experiments of Shandong University.

## Author Contributions

YW performed the experiments, analyzed the data, and wrote the manuscript. YG performed and designed the experiments and analyzed the data. YH and YL performed the experiments and analyzed the data. NL provided guidance for the experiment design and contributed to analyzing and discussing the data. CZ directed the research program, provided guidance and suggestions for the experimental design, analyzed the data, and wrote the manuscript. All authors contributed to the article and approved the submitted version.

## Funding

This work was supported by grants from the National Natural Science Foundation of China (91842305, 81771686), the Shandong Provincial Natural Science Foundation (ZR2020MH260), and the Shandong Provincial Key Research and Development Program (Major Scientific and Technological Innovation Project) (2019JZZY021013).

## Conflict of Interest

The authors declare that the research was conducted in the absence of any commercial or financial relationships that could be construed as a potential conflict of interest.

## Publisher’s Note

All claims expressed in this article are solely those of the authors and do not necessarily represent those of their affiliated organizations, or those of the publisher, the editors and the reviewers. Any product that may be evaluated in this article, or claim that may be made by its manufacturer, is not guaranteed or endorsed by the publisher.
