# Supplementary material for: Murine CXCR3+CXCR6+γδT Cells Reside in the Liver and Provide Protection Against HBV Infection
Source: Front Immunol. 2022 Jan 21;12:757379. doi: 10.3389/fimmu.2021.757379 (PMC8814360; doi:10.3389/fimmu.2021.757379)
Supplement: Supplementary file 1 [file DataSheet_1.docx]

**Supplementary Material**

**Supplementary Figures**

**Supplementary Figure 1**


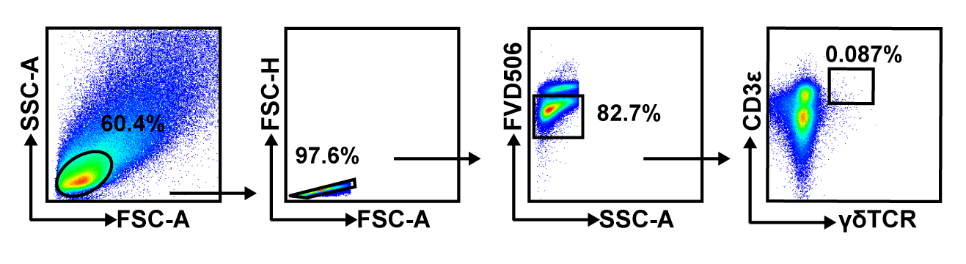


**Supplementary Figure 1.** **The gating strategy of γδT cells.** First, we gated lymphocytes. Second, single-cells were gated through FSC-A and FSC-H. Dead cells were excluded by FVD 506 staining. Next, we gated CD3e^+^ and γδTCR^+^ cells as γδT cells. γδTCR FMO control was used to confirming the gating sites.

**Supplementary Figure 2**


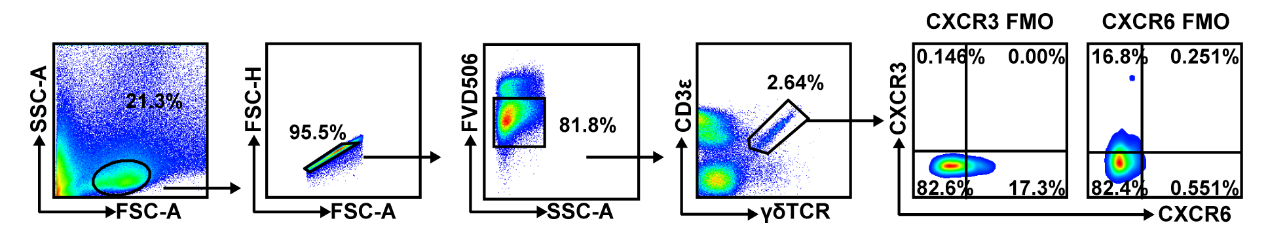


**Supplementary Figure 2. The gating strategy of CXCR3^+^CXCR6^+^ γδT cells.** First, we gated lymphocytes. Second, single-cells were gated through FSC-A and FSC-H. Dead cells were excluded by FVD 506 staining. Next, we gated CD3e^+^ and γδTCR^+^ cells as γδT cells to further analyze the expression of CXCR3 and CXCR6. CXCR3 and CXCR6 FMO controls were used to confirming the gating sites.

**Supplementary Figure 3**


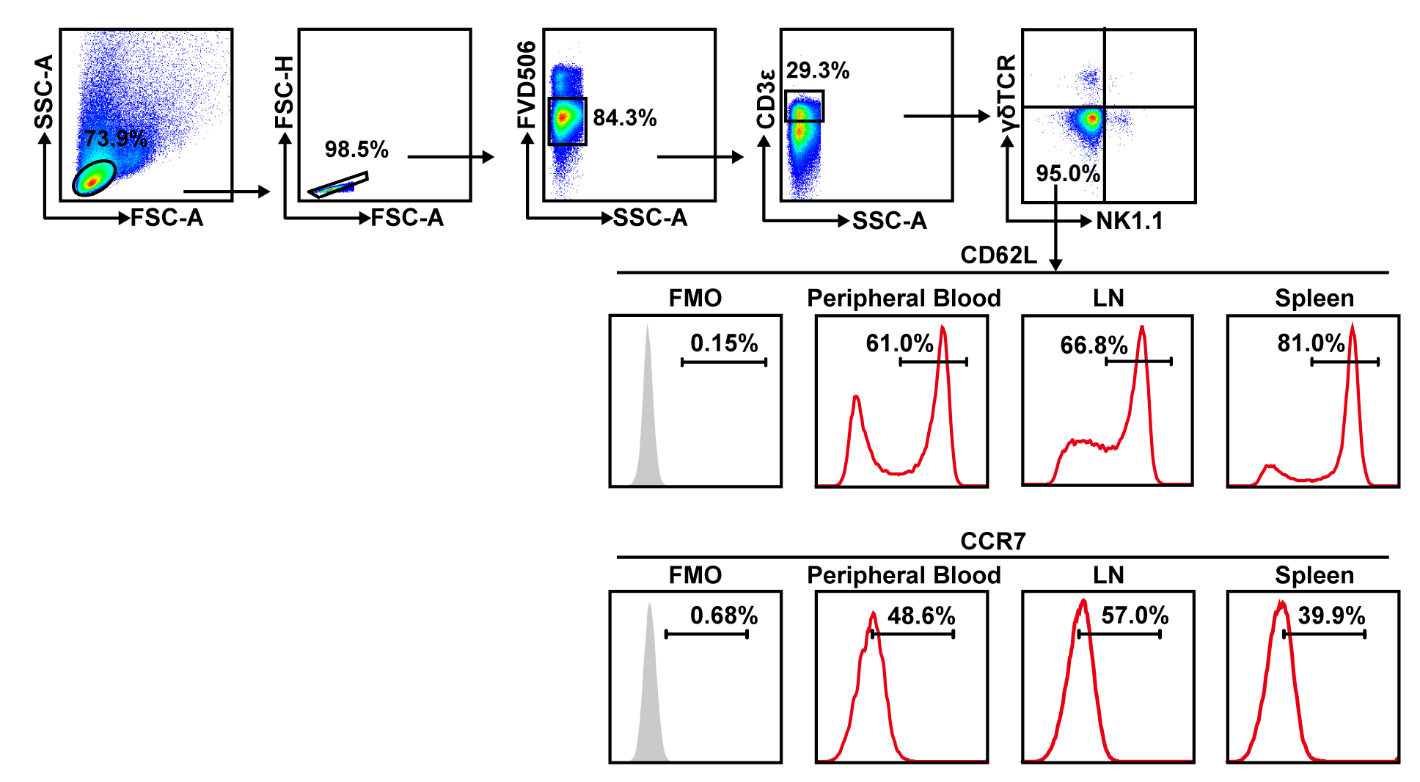


**Supplementary Figure 3. The expression of CD62L and CCR7 on conventional T (CD3^+^TCRγδ^-^NK1.1^-^) cells.** FACS analysis of CD62L and CCR7 expression on conventional T (CD3^+^TCRγδ^-^NK1.1^-^) cells in peripheral blood, lymph nodes and spleen of C57BL/6J mouse.

**Supplementary Figure 4**


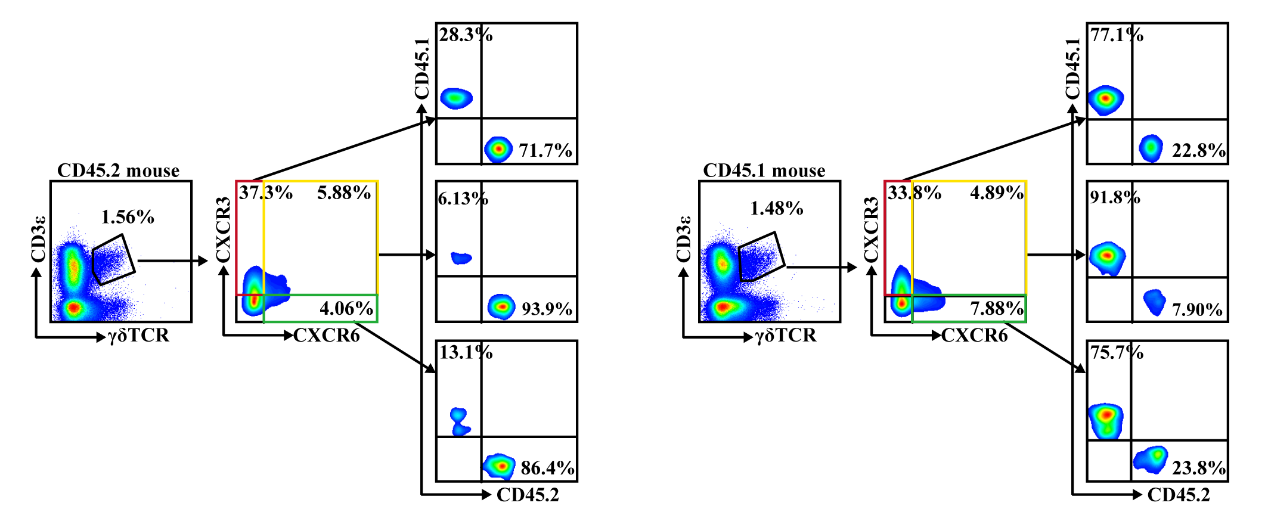


**Supplementary Figure 4.** The host origin (CD45.1^+^ or CD45.2^+^) of hepatic CXCR3^+^CXCR6^-^ γδT cells, CXCR3^+^CXCR6^+^ γδT cells and CXCR3^+^CXCR6^+^ γδT cells was identified by FACS analysis in each mouse in the CD45.1/CD45.2 parabiotic mouse pairs at 14 days post-surgery.

**Supplementary Figure 5**


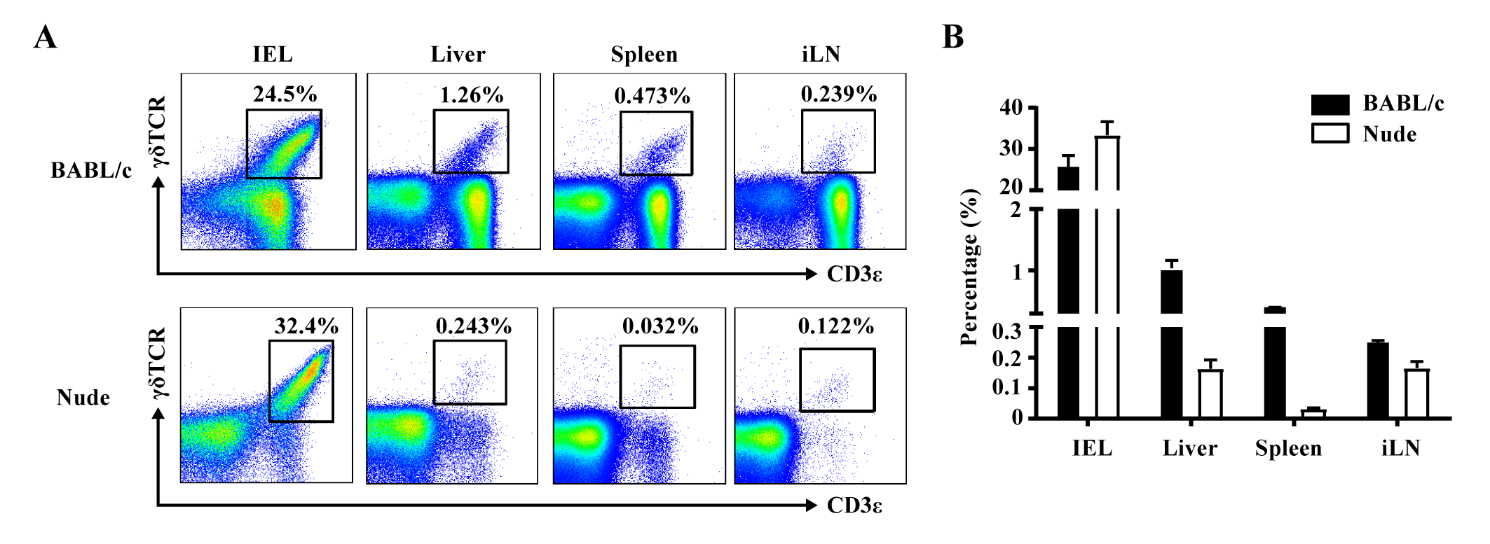


**Supplementary Figure 5. γδT cells exist in the liver of adult athymic nude mice. (A)** Representative FACS plots of γδT cells (CD3^+^TCRγδ^+^) in the indicated organs of BALB/c and nude mice. **(B)** Statistical analysis of γδT cell percentage in the indicated organs of BALB/c and nude mice.

**Supplementary Figure 6**


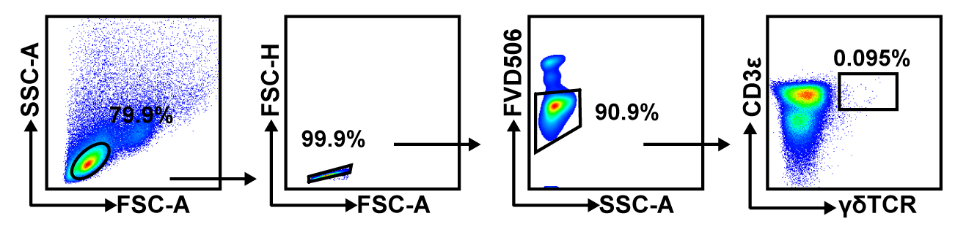


**Supplementary Figure 6.** **The gating strategy of γδT cells.** First, we gated lymphocytes. Second, single-cells were gated through FSC-A and FSC-H. Dead cells were excluded by FVD 506 staining. Next, we gated CD3e^+^ and γδTCR^+^ cells as γδT cells. γδTCR FMO control was used to confirming the gating sites.

**Supplementary Figure 7**


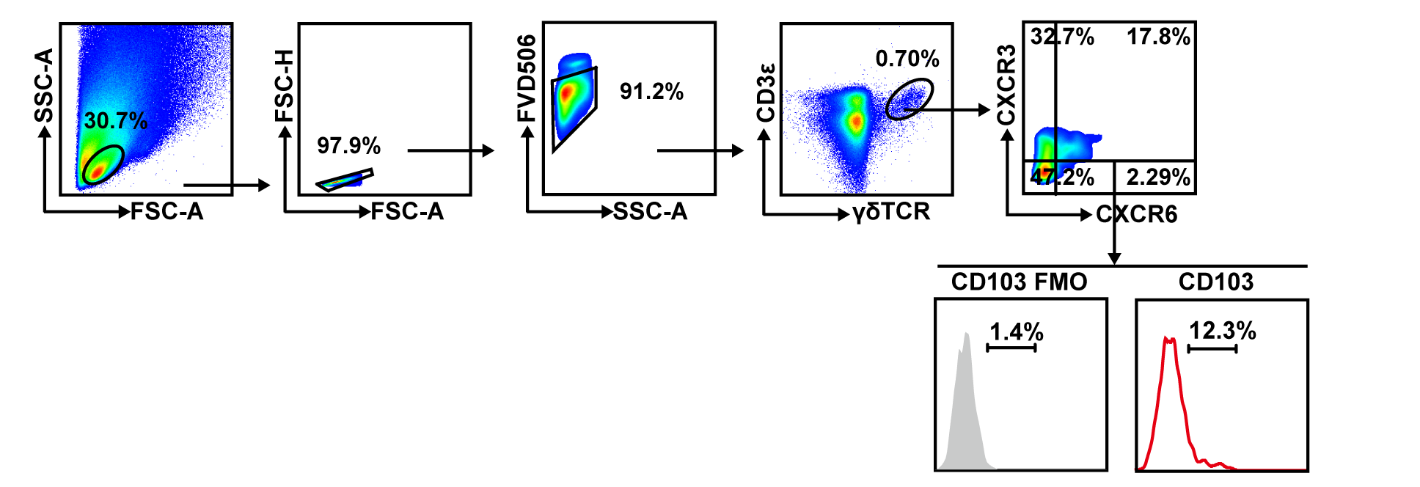


**Supplementary Figure 7.** **The gating strategy of Figure 4.** First, we gated lymphocytes. Second, single-cells were gated through FSC-A and FSC-H. Dead cells were excluded by FVD 506 staining. Next, we gated CD3e^+^ and γδTCR^+^ cells as γδT cells to further analyze the expression of CXCR3 and CXCR6. The FMO control of CD103 was used to confirming the gating site of CD103.

**Supplementary Figure 8**


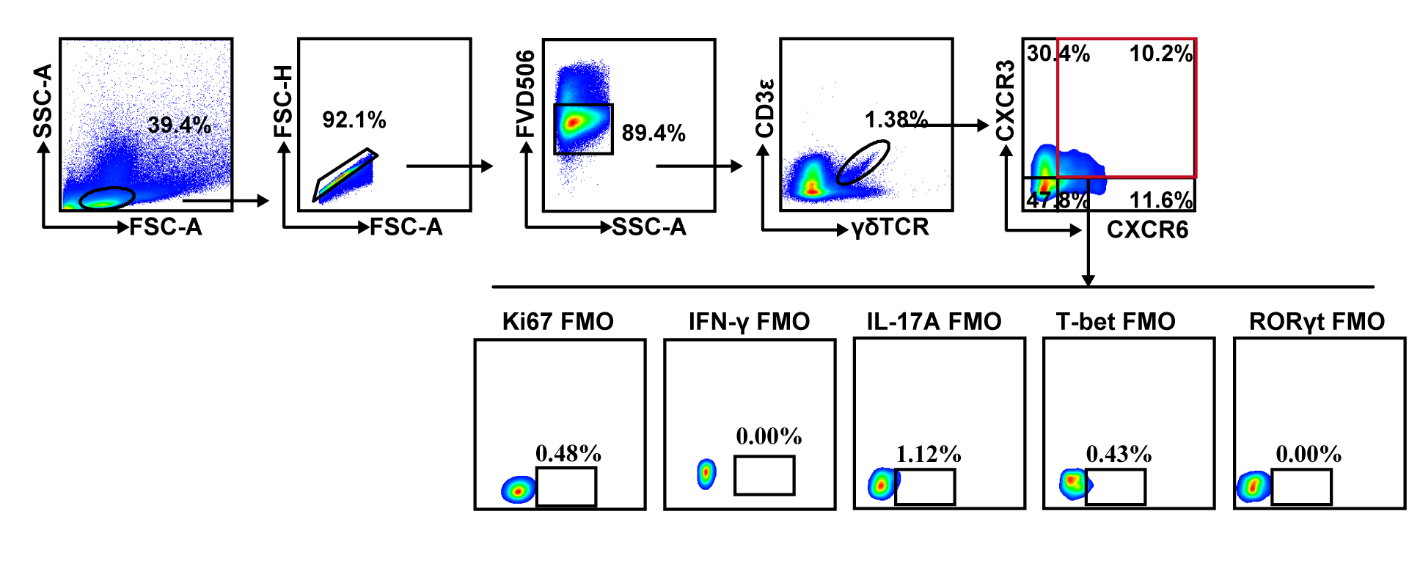


**Supplementary Figure 8. The gating strategy of indicated molecules in Figure 6.** First, we gated lymphocytes. Second, single-cells were gated through FSC-A and FSC-H. Dead cells were excluded by FVD 506 staining. Next, we gated CD3e^+^ and γδTCR^+^ cells as γδT cells and then gated CXCR3^+^ and CXCR6^+^ cells as CXCR3^+^ CXCR6^+^ γδT cells. Then further analyze the expression of Ki67, IFN-γ，IL-17A, T-bet and RORγt.

**Supplementary Tables**

**Supplementary Table 1: Mouse antibody list**

| **Marker** | **Clone** | **Fluor** | **Vendor** |
| --- | --- | --- | --- |
| CD3ε | 145-2C11 | APC/Cyanine7 | Biolegend |
| CD3ε | 145-2C11 | Percp-Cy5.5 | Biolegend |
| TCR γ/δ | GL3 | APC | Biolegend |
| TCR γ/δ | GL3 | FITC | Biolegend |
| CD183 (CXCR3) | CXCR3-173 | PE | Biolegend |
| CD44 | IM7 | APC/Cyanine7 | Biolegend |
| CD27 | LG.3A10 | Brilliant Violet 421 | Biolegend |
| CD314 (NKG2D) | CX5 | PE/Dazzle™ 594 | Biolegend |
| CD122 (IL-2Rβ) | TM-β1 | APC/Cyanine7 | Biolegend |
| IFN-γ | XMG1.2 | PerCP/Cyanine5.5 | Biolegend |
| IFN-γ | XMG1.2 | PE | Biolegend |
| IL-17A | TC11-18H10.1 | PE/Dazzle 594 | Biolegend |
| IL-17A | TC11-18H10.1 | Brilliant Violet 421 | Biolegend |
| PLZF | 9E12 | PE/Cyanine7 | Biolegend |
| CD69 | H1.2F3 | PE/Cyanine7 | Biolegend |
| CD45.2 | 104 | APC/Cyanine7 | Biolegend |
| CD103 | 2E7 | Brilliant Violet 421 | Biolegend |
| CD150 | Q38-480 | BV421 | BD Biosciences |
| CD62L | MEL-14 | V450 | BD Biosciences |
| CD95 | Jo2 | BV421 | BD Biosciences |
| CD45.1 | A20 | PE-CF594 | BD Biosciences |
| ROR gamma (t) | B2D | PE-eFluor 610 | eBioscience |
| T-bet | eBio4B10 | PE-Cyanine7 | eBioscience |
| CXCR6 | 221002 | APC | R&D Systems |

[**Supplementary Table 2**](http://www.sciencedirect.com/science/article/pii/S0304383515006084?via=ihub#ec0010): **Primer sequences used for real-time PCR**

| **Names** | **Forward** | **Reverse** |
| --- | --- | --- |
| *β-actin* | AGAGGGAAATCGTGCGTGAC | CAATAGTGATGACCTGGCCGT |
| *Cxcr3* | GGTTAGTGAACGTCAAGTGCT | CCCCATAATCGTAGGGAGAGGT |
| *Cxcr6* | GAGTCAGCTCTGTACGATGGG | TCCTTGAACTTTAGGAAGCGTTT |
| *Ccr2* | ATCCACGGCATACTATCAACATC | TCGTAGTCATACGGTGTGGTG |
| *Ccr6* | TGGGCCATGCTCCCTAGAA | GGTGAGGACAAAGAGTATGTCTG |
